# Are There Differences between Traumatic and Spontaneous Cervical Artery Dissections?

**DOI:** 10.3390/jcm13154443

**Published:** 2024-07-29

**Authors:** Issa Metanis, Naaem Simaan, Yoel Schwartzmann, Tamer Jubeh, Asaf Honig, Hamza Jubran, Jad Magadle, Jose E. Cohen, Ronen R. Leker

**Affiliations:** 1 Department of Neurology, Hadassah-Hebrew University Medical Center, Jerusalem 9112102, Israel; issa.meta@yahoo.com (I.M.); naaems@ziv.gov.il (N.S.); yoelschwartzmann@gmail.com (Y.S.); tamer0001@hotmail.com (T.J.); asaf.honig2@gmail.com (A.H.); hamzeh-89@hotmail.com (H.J.); j.magadle@gmail.com (J.M.); 2 Department of Neurology, Ziv Medical Center, Safed 1311001, Israel; 3 Department of Neurosurgery, Hadassah-Hebrew University Medical Center, Jerusalem 9112102, Israel; jcohenns@yahoo.com

**Keywords:** dissection, trauma, stroke, outcome

## Abstract

(1) **Background**: Cervical arterial dissections (CeAD) are a common cause of stroke in young adults. CeAD can be spontaneous (sCeAD) or traumatic (tCeAD). Whether CeAD subtypes differ in clinical, radiological, and outcome characteristics remains unexplored. (2) **Methods**: Patients with CeAD were identified and divided between sCeAD and tCeAD. Demographics, clinical features, risk factors, imaging findings, treatments, and outcomes were compared between the groups. Logistic regressions were used to determine characteristics associated with favorable outcome. (3) **Results**: Overall, 154 patients were included (106 sCeAD and 48 tCeAD). Patients with sCeAD were significantly older (mean ± SD 46 ± 12 vs. 35 ± 14, *p* < 0.001) and were more likely to have hyperlipidemia (19% vs. 4%, *p* = 0.016), but other risk factors did not differ. Patients with tCeAD less often had signs of early infarction on imaging (21% vs. 49%, *p* = 0.001) and had lower stroke severity on admission (NIHSS, median, interquartile range [IQR] 0 (0–9) vs. 2 (0–4), *p* = 0.012), but more often had symptomatic intracranial hemorrhages (12.5% vs. 2%, *p* = 0.006). Patients with tCeAD less often had favorable outcomes at 90 days (78% vs. 97%, *p* < 0.001). In the regression analysis, the only variables associated with favorable outcome were age (odds ratio [OR] 1.13, 95% confidence interval [CI] 1.03–1.24), initial stroke severity (OR 0.84, 95% CI 0.73–0.97), degree of vessel stenosis (OR 0.35, 95% CI 0.14–0.83), and involvement of multiple vessels on presentation (OR 0.04, 95% CI 0.02–0.70), whereas dissection subtype was not associated (OR 0.45, 95% CI 0.03–68.80). (4) **Conclusions**: Dissection subtype is not an independent modifier of the chances of attaining functional independence.

## 1. Introduction

Cervical arterial dissections (CeAD) are a common cause of stroke in young adults [1,2]. CeAD can be divided into spontaneous dissections (sCeAD) and dissections secondary to trauma to the neck area (tCeAD) [1,3,4,5,6,7,8,9]. Genetic disorders and other conditions may make the arterial wall more susceptible to minor unremarkable trauma that otherwise would not have caused CeAD, complicating the distinction between tCeAD and sCeAD. Whether traumatic dissections differ from non-traumatic extra-cranial dissections in clinical, radiological, and treatment characteristics remains largely unexplored, with conflicting results reported in the existing data that mostly excluded patients with more severe forms of traumatic neck injury or poly-trauma [4,8,9,10,11]. Only one previous study included patients with more severe forms of trauma and found that tCeAD did not remain a significant predictor of outcome after adjustment for stroke severity [4]. Two other studies that only included patients with mild trauma reported similar results [8,9], but another study reported that patients with tCeAD more frequently had poor outcomes [11]. Furthermore, treatment options may be more restricted in patients with tCeAD in which there may be contraindications to the use of anticoagulants or antiplatelets due to bleeding risks and low hemoglobin levels. Therefore, we aimed to compare data from patients with extracranial traumatic dissection with that obtained in patients with non-traumatic extracranial dissections.

## 2. Materials and Methods

a. Patients: This was a retrospective case series study of patients with extracranial cervical arterial dissections identified from a large cohort of stroke patients admitted to a tertiary teaching hospital between 2013 and 2022. The study was approved by the institutional review board with an exemption from obtaining informed consent due to the retrospective use of anonymized data for this study.

The diagnosis of CeAD was confirmed on imaging studies including CT angiography, MR angiography, or digital subtraction angiography showing evidence of extracranial dissection. Extension of the dissecting segment into the intracranial vasculature was allowed, but patients with isolated intracranial dissections were excluded.

Included patients were divided into sCeAD and tCeAD groups. Spontaneous CeAD was diagnosed when no history of any kind of trauma existed despite an extensive questioning. CeAD secondary to presumed genetic disorders including collagen disorders such as Marfan’s disease, Ehlers Danlos disease, and fibromuscular dysplasia were considered as part of sCeAD. Patients with any history of injury due to direct mechanical trauma to the head or neck region, or any extra ordinarily activity that increased intrathoracic pressure, within 30 days prior to symptom onset, were allocated to the tCeAD group. The allocation to the CeAD group was regardless of the severity of the trauma and in line with the definitions used in the cervical artery dissection and ischemic stroke patients (CADISP) study [4]. We used a slightly modified categorization for the severity of the traumatic injury that was graded as mild (isolated blunt trauma to the neck area without hospitalization due to the trauma itself), moderate (either blunt or perforating significant injury to the neck area that needed hospitalization but without need for surgery), and severe (CeAD as part of poly-trauma involving several organ systems or perforating or blunt neck injury necessitating surgical intervention). We further classified patients with tCeAD according to the Denver grading system for vascular injury [12] (Table A1, grade 1 = irregularity in the vessel lumen with narrowing of <25%, grade 2 = intraluminal thrombus or intimal flap with flow and narrowing >25%, grade 3 = pseudo-aneurysm with flow, grade 4 = vessel occlusion, grade 5 = transection of the vessel with extravasation). Ascertainment of tCeAD was done in real time during the admission by regularly interviewing the patients and family members as per usual standard operation procedures for all patients with CeAD.

b. Clinical data: Data were collected form the electronic medical files including outpatient visits to the neurology and neurosurgery clinics. Collected data included demographics, risk factor profile including the presence of recent infections occurring within the 30 days prior to the diagnosis, stroke characteristics and severity, and imaging findings.

c. Imaging: All patients underwent non-contrast CT scans and CT angiography, and some also had a CT perfusion study. All patients also had an MRI study, unless there were contraindications to MRI. Degree of dissected vessel stenosis on imaging was documented and divided into no stenosis, mild (<50% narrowing), moderate (50–69% narrowing), severe (70–99%), and complete (occlusion). The presence of intimal flaps, intramural hematoma, and pseudo-aneurysms, as well as the radiological appearance of flame-shaped lesions, smooth or irregular narrowing, and double lumen in the involved vessel were also documented.

d. Imaging and clinical outcomes: Recanalization of the dissected vessel was assessed with follow-up vascular imaging including duplex ultrasound or CT angiography. Follow-up imaging was obtained at or before the first follow-up visit to the stroke clinic, usually within 30 days of discharge, but in some cases, it was longer. Recanalization was classified as either present or absent, and the presence or degree of residual stenosis was not captured during visits.

We repeated the same analyses for patients with a final diagnosis of stroke during the same admission as the CeAD. This set of analyses included patients that presented with imaging and clinical symptoms suggestive of stroke, as well as those who developed clinical or imaging evidence of stroke during the same admission. We also collected information on the treatments given and outcome parameters.

Favorable outcome was defined as modified Rankin score (mRS) of ≤2 at 90 days after cervical arterial dissection and stroke. Survival data were also studied, as were rates of recurrent stroke and recurrent dissection. The rates of symptomatic intracerebral hemorrhage (sICH), defined as the presence of intracerebral hemorrhage associated with a change of 4 points on the NIHSS scale, were also recorded as an outcome measure.

e. Statistical analysis: Statistical analysis was performed using the SPSS 29 software (IBM USA). The X^2^ test was used to explore the link between qualitative variables. Student’s *t*-test and Fisher’s exact test were used to compare continuous parametric variables, and the Mann–Whitney test and median tests were used for nonparametric testing. *p* < 0.05 was considered significant. Regression analyses including variables that yielded a *p* value of <0.1 on the univariable analyses were performed with the mandatory inclusion of CeAD subtype in order to study whether CeAD subtype influenced the chances for achieving favorable functional outcomes at 90 days post-stroke. In a separate analysis among patients that had a stroke, we included variables that are well known to be associated with outcomes including age, NIHSS at presentation, and pre-existing disability, in addition to dissection type.

## 3. Results

Overall, 154 patients with CeAD were identified and included in the study. Of those, 106 (69%) patients were diagnosed with sCeAD (57% men), and 48 (31%) patients (69% men) with tCeAD. The median age was 44 (interquartile range [IQR] 35–52). The frequency of headache or isolated Horner sign on presentation were similar between the groups (30% and 7%, respectively). Focal neurological deficits lasting for more than 24 h or the presence of focal brain injury on imaging were present in 82 patients overall (61% males, median age 46 [IQR 38.75–55]). Of those, 68 (83%) had clinical and/or imaging findings of stroke on admission, and in the remaining 14 (17%), stroke symptom/signs became apparent during the acute hospitalization.

Of the tCeAD patients, 16 (33%) had mild traumatic injury, 11 (23%) were diagnosed with moderate injury, and 21 (44%) were diagnosed with severe injury. tCeAD patients were further categorized according to the Denver scale for cervical injury. Of the 48 patients with tCeAD, 9 (19%) were classified as grade 1, 12 (25%) as grade 2, 12 (25%) as grade 3, 11 (23%) as grade 4, and 4 (8%) as grade 5.

Baseline characteristics: In the analyses of the entire study population, patients with sCeAD were significantly older (mean ± SD 46 ± 12 vs. 35 ± 14, *p* < 0.001) and were more likely to have hyperlipidemia (19% vs. 4%, *p* = 0.016). However, the frequency of other traditional stroke baseline risk factors and site of vessel occlusion did not differ between the groups (Table 1). Patients with sCeAD more often presented with stroke both clinically (56% vs. 27%; *p* = 0.001) and on imaging upon admission (49% vs. 21%; *p* = 0.001). In the entire cohort, admission neurological deficits were more severe in patients with sCeAD (NIHSS median [IQR], 2 (0–4) vs. 0 (0–9), *p* = 0.012). However, among patients diagnosed with stroke, the initial stroke severity tended to be higher in patients with tCeAD (NIHSS median [IQR], 9 (0–21.75) vs. 3 (1–6); *p* = 0.068; Table A1).

Imaging data: The most commonly affected arterial segments were the extracranial carotid arteries (*n* = 84, 54%), whereas only 48 patients (31%) had vertebral artery dissections (Table 1). Of note, 30 patients (20%) suffered from multi-vessel dissections that were more common in patients with tCeAD (33% vs. 13%, *p* = 0.003). The results were similar when limiting the analysis to inclusion of only patients with stroke (Table A2). The severity of vessel narrowing was significantly milder in patients with tCeAD, and more patients with sCeAD had severe stenosis (*p* = 0.013, Table 1).

Analysis of the imaging findings showed that intimal flaps were more commonly seen among patients with tCeAD (34% vs. 16%, *p* = 0.012), whereas long irregular stenoses were more common in the sCeAD group (51% vs. 23%, *p* = 0.001). However, other imaging findings including the presence of pseudo aneurysms, elongated tapering, double lumen, mural hematoma, and vessel occlusions did not differ between the groups (Table 1). When limiting the analyses to only patients with stroke, the only finding that differed between the groups was that of elongated narrowing, which was more commonly seen in the sCeAD group (51% vs. 14%; *p* = 0.011).

Treatment: Systemic thrombolysis use was significantly higher in patients with sCeAD (8.5% vs. 0%, *p* = 0.037). However, the frequency of stent placement did not differ between the groups (Table 1). Initial treatment with low-molecular-weight heparin was numerically more common in the tCeAD patients, but the difference between the groups did not reach significance (42% vs. 34%, *p* = 0.38). When limiting the analysis to patients with stroke, the difference between the groups became significant (71% vs. 34%; *p* = 0.010). Similar proportions of patients were treated with antiplatelets for the long run (76% and 79% in the sCeAD and tCeAD groups, respectively).

Follow-up vascular and clinical outcomes: Follow-up vascular imaging studies were available for 142 patients (92%) and mostly consisted of CT angiographies (87% and 97% in tCeAD and sCeAD, respectively). The median time for assessment of recanalization stratus was 30 days (IQR 0–90 days) and did not differ between the two groups. There were no statistically significant differences in the rates of recanalization among the groups (53% vs. 68% in sCeAD vs. tCeAD, respectively; *p* = 0.081).

**Figure 1:**
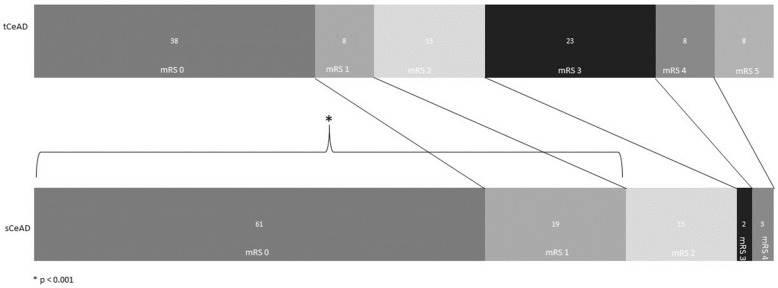
Functional outcomes in patients with CeAD and stroke. Bar graph plots depicting functional outcomes in patients with CeAD and stroke 90 days post-admission. Numbers represent percentages of patients in each category. mRS—modified Rankin Scale, sCeAD—spontaneous cervical artery dissection, tCeAD—traumatic cervical artery dissection.

Most patients were independent prior to the CeAD, and only two patients had mild disability prior to stroke (mRS ≥ 3, 1%). Median follow-up was 12 months (IQR 10.75–19). Outcome data at 90 days post presentation were available for 146 of the included patients (95% overall, 100/106 in the sCeAD group and 46/48 in the tCeAD group), and only eight patients were lost to follow-up. Outcomes significantly differed between the groups, both at discharge and at 90 days (Table 2). The rates of any ICH and symptomatic ICH were significantly higher in tCeAD patients compared to those with sCeAD 17% vs. 5% and 12.5% vs. 2%; *p* = 0.013 and *p* = 0.006, respectively). The results remained significant for the differences in the rates of sICH when the analysis was limited to inclusion of only patients with stroke (21% vs. 3%; *p* < 0.001, Table A2). The rates of functional independence at 90 days were significantly higher among sCeAD patients in the entire cohort analysis (97% vs. 78%, *p* < 0.001, Table 2). Similar results were seen when the analysis was limited to include only patients with stoke (95% vs. 54%; *p* < 0.001, Figure 1 and Table A3). The occurrence of recurrent stroke and recurrent dissections were very low within the first 90 days, with no significant differences between the groups (Table 2).

In order to explore whether dissection subtype was associated with the likelihood of favorable outcomes, we compared patients with favorable outcomes (*n* = 133) to those with unfavorable outcomes (*n* = 13). In the univariate analysis (Table 3), patients with favorable outcomes were significantly older (mean ± sd 44 ± 13 vs. 35 ± 14, *p* = 0.024) and less likely to have dissections involving multiple arteries (16% vs. 54%, *p* = 0.001). Analysis of the imaging characteristics (Table 3) showed that elongated vessel stenosis patterns were more common in patients with favorable outcomes (46% vs. 15%, *p* = 0.034), whereas flame-shaped appearance and vessel occlusions were less common in patients with favorable outcomes (23% vs. 69% and 24% vs. 62%, *p* < 0.001 for both). Other imaging markers for CeAD upon arrival did not differ between the groups (Table 3). Patients with favorable outcomes had lower NIHSS scores on presentation (median, (IQR) 0 (0–3) vs. 11 (4.25–11.75) *p* = 0.027) and less often suffered from sICH (2% vs. 23%, *p* < 0.001). Patients that received stent placements were less likely to achieve favorable outcomes (34% vs. 62%, *p* = 0.047). However, the risk factor profiles, as well as use of tPA or vessel recanalization, did not differ between the groups. Similar results were obtained when repeating these analyses in the subgroup of 78 of 82 (95%) patients diagnosed with stroke for whom follow-up data were available.

When we eliminated patients with mild trauma from the analysis, we found that higher degrees of trauma severity were associated with lower chances for favorable outcomes (67% vs. 97%, *p* < 0.001).

In the regression analysis controlling for age, stroke severity on admission, involvement of multiple vessels, degree of vessel stenosis, severity of the traumatic injury, the presence of sICH, and dissection type (Table 4), the only variables that remained associated with outcome were age (odds ratio [OR] 1.13, 95% confidence interval [CI] 1.03–1.24), stroke severity (OR 0.84, 95% CI 0.73–0.97), degree of stenosis (OR 0.35, 95% CI 0.14–0.83), and involvement of multiple vessels on presentation (OR 0.38, 95% CI 0.02–0.71). Dissection subtype was not associated with outcome in this model (OR 0.45, 95% CI 0.03–68.80).

On a second regression analysis limited to patients with stroke and controlling for age, admission NIHSS and pre-existing disability dissection type were not associated with the chances for favorable outcomes (Table 5).

## 4. Discussion

The main findings of the current study are that patients with tCeAD less often achieve functional independence at 90 days, despite of being younger and presenting less often with stroke symptoms or signs. Patients with tCeAD had higher sICH rates, which may account for the lower rates of favorable outcomes observed in this population. Furthermore, patients with tCeAD often had severe traumatic injury involving several organ systems. Indeed, we found a significant correlation between trauma severity and outcome. Therefore, at least in some cases, the less favorable functional outcomes could be attributable to the effects of multi-organ injury and trauma severity. However, in a logistic regression model incorporating several clinical and radiological parameters yielding a significant or near significant difference in the univariate analysis, the subtype of CeAD was not associated with outcomes. This may indicate that the subtype of CeAD is a less important modifier of outcome when compared to stroke severity, age, and the severity and breadth of the trauma leading to CeAD.

Interestingly, the only imaging markers that were associated with dissection subtype were intimal flaps that were more commonly seen among patients with tCeAD and elongated irregular stenoses that were more common in the sCeAD group. All other imaging findings including the presence of pseudo aneurysms, elongated tapering, double lumen, mural hematoma, and vessel occlusions did not discern between the dissection subtype groups. As expected, patients with vessel occlusions on imaging were less likely to have favorable outcomes. Surprisingly, we found that elongated narrowing of the dissected vessels was associated with higher chances of favorable outcomes. One plausible explanation for this finding is a more frequent use of stenting in these patients. However, we found that the rates of favorable outcome did not depend on stent placement in these patients. Therefore, we speculate that the higher rates of favorable outcomes in these patients could be related to the fact that the artery was stenotic but not occluded, and therefore not necessarily leading to stroke, which would have been correlated with a higher likelihood of poor outcomes. Indeed only 57% of patients with elongated stenosis had a stroke.

Importantly, in patients that required vessel recanalization as part of their treatment, time from symptom onset to such treatment did not differ between the groups. As expected, tPA use was limited to only sCeAD patients [1,13,14], which may have contributed to the better outcomes observed in sCeAD patients via achievement of earlier recanalization mediated by tPA. Interestingly, endovascular intervention with stent placement [15,16,17,18] was equally used as a therapeutic modality in tCeAD and sCeAD patients in the current series. Surprisingly, stent placement was more frequent among patients with poor outcomes. This may be explained by the higher likelihood of stent placement among patients with stroke symptoms and vessel occlusion or near occlusion upon presentation.

Interestingly, a large proportion of patients were initially treated with low-molecular-weight heparin, and most patients received antiplatelets in both groups, suggesting that at least some of the patients with tCeAD were considered as having low risk for bleeding complications. Short- or long-term treatment modalities with antiplatelets or anticoagulants were not associated with outcome.

We were able to identify only several studies comparing tCeAD and sCeAD [4,8,9,10,11], and most prior studies only included either sCeAD or tCeAD patients [5,6,14,19,20,21].

In agreement with the current results, a study exploring a large cohort of CeAD patients found that tCeAD did not remain a significant predictor of outcome after adjustment for stroke severity [4]. This is the only study we could identify that included patients with severe traumatic injury, including patients with poly-trauma. However, the percentage of patients with severe trauma included in that study was lower compared to the current study, which may have resulted in an underestimation of the effect of trauma severity on outcomes. Another study compared 120 sCeAD patients to 28 patients with mild tCeAD [8]. In agreement with the current results, this study showed that patients with tCeAD were significantly younger than those with sCeAD and more often had dissections involving multiple arterial sites. In that study outcome did not significantly differ between the tCeAD and sCeAD patients, but patients with severe trauma were excluded [8]. A smaller single-center study that included 47 patients found a lower incidence of hyperlipidemia and focal signs among patients with tCeAD that were significantly younger than those with sCeAD [9]. No significant differences in outcomes were noted between the groups [9]. In contrast to these data, one prior study that only included patients with vertebral artery dissections found that patients with tCeAD more frequently had poor outcomes [11]. However, the authors did not perform regression analyses to assess whether the effects of dissection subtype on outcomes are maintained after adjusting for confounding variables.

Similar to prior studies, we also found an association of trauma severity with likelihood of stroke development [6]. Furthermore, as noted above, patients with multi-organ trauma or severe neck trauma were more likely to have poor outcomes compared to those without these attributes, probably reflecting, at least in part, the effects of stroke on outcomes.

It is interesting to note that arterial stiffness has been shown to be associated with cervical arterial dissection [22] and cardiovascular events [23]. Unfortunately, we did not have data on arterial stiffness and therefore could not compare arterial stiffness between the study groups.

In summary, and taken together with the current set of results that included a large number of patients with severe trauma, it appears that while patients with tCeAD more often have poor outcomes compared to sCeAD patients, outcomes depend more on the severity of stroke and trauma then on the mechanism responsible for CeAD.

The strengths of the current study include the all-inclusive design, used in order to include as many patients with CeAD as possible. Notably, a relatively large proportion of our tCeAD patients had more severe degrees of traumatic neck injury, which may better reflect the daily practice in a major teaching trauma center hospital. Our study also had a high rate of follow-up availability at 95%, lending further support to the validity of the results.

The current study also has several limitations. The retrospective design, as well as the single-center setting are potential sources of bias. However, all patients with CeAD were included, and we had no pre-specified exclusion criteria other than isolated intracranial dissections. Second, our patients were all seen in a tertiary, teaching, level one trauma center, and therefore the generalization of the current results to other medical setups is not clear. Third, we used the mRS at 90 days post-diagnosis as the primary outcome measure for the study, but this scale may not be the optimal assessment tool for patients with traumatic injury. However, using a tool that is more specific to trauma would have been impractical for patients with sCeAD. Another limitation of the current study is that we only had a limited number of unfavorable outcomes. Therefore, the results of our regression analyses implying that dissection subtype was not associated with outcomes should be interpreted with caution.

## 5. Conclusions

In conclusion, patients with tCeAD less often have favorable outcomes compared to patients who suffer with sCeAD from a univariate analysis. However, in a multivariate analysis controlling for stroke severity, trauma severity, age, and the presence of vessel occlusion, dissection subtypes were not associated with outcomes. These results are in agreement with most available prior data. However, patients with tCeAD that suffered a stroke did present with higher stroke severity and had higher rates of sICH and multi-territorial involvement, and their outcomes may also have been affected by the multi-organ trauma sustained by some tCeAD patients. This may suggest that a subset of patients with tCeAD are at higher risk of sustaining unfavorable outcomes. The results of the current study should trigger larger prospective multicenter studies comparing tCeAD to sCeAD patients in order to explore whether certain subgroups of patients are more likely to suffer poor outcomes and could be offered different treatment approaches such as endovascular therapy applied early in the course of the disease.

## Figures and Tables

**Table 1:** Baseline characteristics of patients with spontaneous and traumatic dissection.

Variable/Group	Spontaneous (*n* = 106)	Traumatic (*n* = 48)	*p*-Value
Age (±SD)	46 (±12)	35 (±14)	<0.001
Male gender (%)	43 (57)	23 (70)	0.225
Hypertension (%)	20 (27)	5 (15)	0.191
Diabetes (%)	7 (9)	1 (3)	0.249
Atrial fibrillation (%)	3 (4)	0 (0)	0.244
Hyperlipidemia (%)	13 (17)	1 (3)	0.042
Smoking (%)	21 (28)	13 (39)	0.240
Prior stroke (%)	3 (4)	0 (0)	0.244
Recent infection (%)	9 (12)	2 (6)	0.347
Migraine (%)	4 (6)	0 (0)	0.133
Pregnancy (%)	2 (3)	0 (0)	0.344
Post-partum (%)	3 (4)	0 (0)	0.244
Stroke symptoms on admission (%)	59 (56)	13 (27)	0.001
Admission NIHSS (median IQR)	2 (0–4)	0 (0–9)	0.012
Early infarct on imaging (%)	52 (49)	10 (21)	0.001
Involved vessels:			
Carotids only (%)	62 (58)	22 (46)	0.144
Vertebrals only (%)	33 (31)	15 (31)	0.988
Multiple vessels (%)	14 (13)	16 (33)	0.003
Stenosis (%)			0.013
No stenosis	21 (20)	20 (42)	
<50%	5 (5)	4 (8)	
50–69%	12 (11)	1 (2)	
70–99%	33 (31)	8 (17)	
100%	35 (33)	15 (31)	
Radiological findings (%)			
Intimal flap	17 (16)	16 (34)	0.012
Pseudo-aneurism	32 (30)	13 (28)	0.784
Mural hematoma	18 (17)	13 (27)	0.148
Flame-shaped occlusion	29 (27)	13 (27)	0.972
Double lumen	5 (5)	4 (8)	0.376
Elongated irregular narrowing	55 (51)	11 (23)	0.001
Time from onset to recanalization treatment (minutes, median IQR)	1410 (205–20610)	1680 (540–4320)	0.573
tPA (%)	9 (8.5)	0 (0)	0.037
Stent placement (%)	37 (40)	19 (35)	0.576

NIHSS—National Institutes of Health Stroke Scale, tPA—tissue plasminogen activator.

**Table 2:** Outcomes and treatment.

Variable/Group	Spontaneous (*n* = 106)	Traumatic (*n* = 48)	*p*-Value
Stroke diagnosis at discharge (%)	68 (64)	14 (29)	<0.001
Any ICH (%)	5 (5)	8 (17)	0.013
sICH (%)	2 (2)	6 (12.5)	0.006
Recurrent stroke * (%)	1 (1)	3 (10)	0.081
Recurrent dissection ** (%)	2 (2)	1 (3)	1.00
Recanalization * (%)	40 (49)	22 (67)	0.082
Initial LMWH (%)	36 (34)	20 (42)	0.379
Long-term anti-platelets (%)	81 (76)	38 (79)	0.706
Long-term oral anticoagulants (%)	7 (7)	0 (0)	0.068
mRS 90 (median, IQR)	0 (0–2)	0 (0–2)	0.188
mRS 90 ≤ 2 *** (%)	97 (97%)	36 (78%)	<0.001

ICH—intracerebral hemorrhage, LMWH—low-molecular-weight heparin, mRS—modified Rankin Scale, IQR—interquartile range, sICH—symptomatic intracerebral hemorrhage. * *n* = 100, ** *n* = 116, *** *n* = 146.

**Table 3:** Comparison of patients with favorable and unfavorable outcomes.

Variable/Group	Favorable Outcome (*n* = 133)	Unfavorable Outcome (*n* = 13)	*p*-Value
Age (±SD)	44 (±13)	35 (±14)	0.024
Male gender (%)	77 (58)	10 (77)	0.182
Hypertension (%)	29 (22)	2 (15)	0.589
Diabetes (%)	10 (7.5)	0 (0)	0.306
Atrial fibrillation (%)	4 (3)	0 (0)	0.526
Ischemic heart disease (%)	3 (2)	0 (0)	0.584
Hyperlipidemia (%)	19 (14)	1 (8)	0.509
Smoking (%)	34 (26)	4 (31)	0.683
Prior stroke (%)	3 (2)	1 (8)	0.252
Infection (%)	12 (9)	1 (8)	0.784
Cough (%)	12 (13)	1 (10)	0.784
Early infarct on imaging (%)	52 (39)	5 (38.5)	0.964
Involved vessels:			
Carotids only (%)	73 (55)	5 (38.5)	0.257
Vertebrals only (%)	43 (32)	3 (23)	0.493
Multiple vessels (%)	22 (16.5)	7 (54)	0.001
Radiological findings (%)			
Intimal flap	28 (21)	3 (25)	0.749
Pseudo-aneurism	38 (29)	3 (25)	0.769
Mural hematoma	27 (20)	2 (15)	0.672
Long stenosis	61 (46)	2 (15)	0.034
Double lumen	9 (7)	0 (0)	0.333
Occlusion above bifurcation	32 (24)	8 (61.5)	0.004
Admission NIHSS	3 ± 6	14 ± 13	<0.001
tPA (%)	9 (7)	0 (0)	0.333
Stent placement (%)	45 (34)	8 (61.5)	0.047
Any ICH (%)	8 (6)	3 (23)	0.026
sICH (%)	3 (2)	3 (23)	<0.001
Recurrent dissection (%)	2 (2)	1 (8)	0.130
Recanalization (%)	54 (52)	6 (67)	0.411
Dissection type (sCeAD, %)	97 (73)	3 (23)	<0.003

ICH—intracerebral hemorrhage, NIHSS—National Institutes of Health Stroke Scale, tPA—tissue plasminogen activator, sICH—symptomatic intracerebral hemorrhage.

**Table 4:** Factors associated with favorable outcome at 90 days post-stroke on logistic regression.

Variable	OR	95% CI	
Age (per year)	1.13	1.03	1.24
Trauma severity (per degree)	0.44	0.07	2.82
Severity of vessel stenosis	0.35	0.15	0.83
Multiple arteries	0.04	0.002	0.70
sICH	1.53	0.03	74.09
Admission NIHSS	0.84	0.73	0.97
Dissection type *	0.45	0.03	60.81

NIHSS—National institutes of Health Stroke Scale, sICH—symptomatic intracranial hemorrhage, * spontaneous dissection.

**Table 5:** Factors associated with favorable outcome at 90 days post-stroke on logistic regression.

	OR	95% C.I.		*p*-Value
Age	1.073	0.974	1.180	0.152
Admission NIHSS	0.872	0.774	0.981	0.023
Dissection type *	7.269	0.792	66.676	0.079
Pre-stroke mRS	2.170	0.428	11.002	0.350

* Spontaneous. NIHSS—National Institutes of Health Stroke Scale, mRS—modified Rankin Score.

## Data Availability

Full data are available following a formal request and in compliance with state regulations.

## Appendix A

**Table A1:** Denver “grading scale for blunt carotid injury”.

Grade	Description
Grade I	Arteriographic appearance of irregularity of the vessel wall or a dissection/intramural hematoma with less than 25% luminal stenosis
Grade II	Intraluminal thrombus or raised intimal flap is visualized, or dissection/intramural hematoma with 25% or more luminal narrowing
Grade III	Pseudoaneurysm
Grade IV	Vessel occlusion
Grade V	Transection with free extravasation

**Table A2:** Baseline characteristics in patients with CeAD and stroke.

Variable/Group	Spontaneous (*n* = 68)	Traumatic (*n* = 14)	*p*-Value
Age (±SD)	47 (±10)	34 (±15)	0.032
Male gender (%)	40 (59)	10 (71)	0.379
Hypertension (%)	18 (26)	3 (21)	0.694
Diabetes (%)	9 (13)	0 (0)	0.149
Atrial fibrillation (%)	4 (6)	0 (0)	0.352
Hyperlipidemia (%)	13 (19)	1 (7)	0.278
Smoking (%)	17 (25)	5 (36)	0.410
Prior stroke (%)	2 (3)	1 (7)	0.446
Recent infection (%)	7 (10)	0 (0)	0.226
Migraine (%)	0 (0)	0 (0)	1.00
Pregnancy (%)	1 (1.5)	0 (0)	0.648
Stroke symptoms on admission (%)	59 (56)	13 (27)	0.001
Admission NIHSS (median IQR)	3 (1–6)	0 (0–21.75)	0.068
Infarct on admission imaging (%)	46 (68)	8 (57)	0.450
Involved vessels:			
Carotids only (%)	39 (57)	7 (50)	0.614
Vertebrals only (%)	22 (32)	3 (24)	0.419
Multiple vessels (%)	8 (12)	6 (43)	0.005
Stenosis (%)			0.589
No stenosis	9 (13)	3 (21)	
<50%	2 (3)	0 (0)	
50–69%	6 (9)	1 (7)	
70–99%	22 (34)	2 (14)	
100%	29 (43)	8 (57)	
Radiological findings (%)			
Pseudo-aneurism	18 (27)	7 (50)	0.082
Mural hematoma	16 (24)	6 (43)	0.137
Elongated stenosis	35 (52)	2 (14)	0.011
Double lumen	3 (4)	1 (7)	0.666
Occlusion above bifurcation	24 (35)	7 (50)	0.301
Long tapering	0 (0)	1 (7)	0.027
Flame-shaped occlusion	23 (34)	8 (57)	0.101
Time from onset to recanalization treatment (minutes, median IQR)	547 (185–9720)	540 (282–2070)	0.477
tPA (%)	9 (13)	0 (0)	0.149
Stent placement (%)	31 (46)	10 (71)	0.078

NIHSS—National Institutes of Health Stroke Scale, tPA—tissue plasminogen activator.

**Table A3:** Treatments and outcomes in patients with CeAD and stroke.

Variable/Group	Spontaneous (*n* = 68)	Traumatic (*n* = 14)	*p*-Value
Any ICH (%)	4 (6)	3 (21)	0.058
sICH (%)	2 (3)	3 (21)	0.008
Recurrent stroke (%)	0 (0)	0 (0)	1.00
Recurrent dissection (%)	1 (1)	1 (7)	0.243
Recanalization (%)	33 (53)	11 (79)	0.083
Initial LMWH (%)	23 (34)	10 (71)	0.010
Long-term anti-platelets (%)	53 (78)	11 (79)	0.959
Long-term oral anticoagulants (%)	29 (43)	9 (64)	0.139
mRS 90 ≤2 *** (%)	67 (95)	7 (54)	<0.001

ICH—intracerebral hemorrhage, LMWH—low-molecular-weight heparin, mRS—modified Rankin Scale, sICH—symptomatic intracerebral hemorrhage. ***—data available for 78 of 82 patients.
